# Invited Commentary: Treatment Drop-in—Making the Case for Causal Prediction

**DOI:** 10.1093/aje/kwab030

**Published:** 2021-02-17

**Authors:** Matthew Sperrin, Karla Diaz-Ordaz, Romin Pajouheshnia

**Keywords:** counterfactual causal inference, risk prediction, treatment drop-in

## Abstract

Clinical prediction models (CPMs) are often used to guide treatment initiation, with individuals at high risk offered treatment. This implicitly assumes that the probability quoted from a CPM represents the risk to an individual of an adverse outcome in absence of treatment. However, for a CPM to correctly target this estimand requires careful causal thinking. One problem that needs to be overcome is treatment drop-in: where individuals in the development data commence treatment after the time of prediction but before the outcome occurs. In this issue of the *Journal*, Xu et al. (*Am J Epidemiol*. 2021;190(10):2000–2014) use causal estimates from external data sources, such as clinical trials, to adjust CPMs for treatment drop-in. This represents a pragmatic and promising approach to address this issue, and it illustrates the value of utilizing causal inference in prediction. Building causality into the prediction pipeline can also bring other benefits. These include the ability to make and compare hypothetical predictions under different interventions, to make CPMs more explainable and transparent, and to improve model generalizability. Enriching CPMs with causal inference therefore has the potential to add considerable value to the role of prediction in healthcare.

## Abbreviations

CPM: clinical prediction model

***Editor’s note:** The opinions expressed in this article are those of the authors and do not necessarily reflect the views of the* American Journal of Epidemiology.

Clinical prediction models (CPMs) predict the risk of adverse outcomes for individuals, such as the future risk of a cardiovascular event (e.g., acute myocardial infarction) for an individual in primary care (1). CPMs are commonly used to guide decisions concerning intervention, such as initiating treatment—for example, statin initiation for individuals at high cardiovascular risk (2). Such a use assumes, explicitly or implicitly, that the prediction issued by a CPM is a treatment-naive prediction—that is, the (hypothetical) risk of an outcome if the individual does not commence treatment (3). Constructing CPMs that estimate this hypothetical risk is nontrivial, not least because of treatment drop-in, where individuals in the development data set commence treatment after baseline but before occurrence of an outcome (4). Deriving a CPM that correctly estimates treatment-naive risk, in the presence of treatment drop-in, is challenging because individuals do not commence treatment at random (3, 5).

In an excellent contribution in this issue of the *Journal*, Xu et al. (6) recommend a pragmatic approach to handling treatment drop-in, illustrated with the example of statin initiation in cardiovascular CPMs. Their proposal is to take the relative risk reduction for statins, estimated in randomized controlled trials, and fix the coefficient for statins to this value, with statins treated as a time-dependent variable in the model. A similar idea to this has been proposed before in the context of treatment drop-in in clinical trials (7), and indeed the general idea of using external data sets to make adjustments has also been proposed in the multiple imputation literature (8).

Previous approaches to address treatment drop-in for CPMs have estimated the effect of treatment from the same data as the development data used for the CPM—using causal inference techniques such as inverse probability weighting (9) or marginal structural models (3). Xu et al.’s approach avoids requiring the usual assumptions when making causal inference with observational data (such as conditional exchangeability and positivity). However, it introduces assumptions concerning the generalizability of the trial estimate and also disregards the uncertainty in the trial estimate. Nevertheless, the simplicity of the approach is a substantial advantage: only requiring the handling of time-dependent covariates when modeling. Moreover, the approach could be readily extended to incorporate approaches for generalizing estimated treatment effects from a randomized controlled trial to broader populations (10, 11). Uncertainty could also be considered, for example, through draws from the posterior distribution of the causal effect size.

As soon as one entertains the need for “treatment naive-risk,” one is targeting estimands that require causal reasoning to estimate well, given that they are hypothetical or counterfactual predictions (12). We note that hypothetical prediction aims at answering “what if” questions about the future, while counterfactual prediction requires contemplating states contrary to what has truly happened, and this difference can be important (13). Here we will use the less-specific term causal prediction. Failure to recognize when a clinical question requires methods for causal prediction can lead to the development of a model that targets the wrong estimand, such as a “treated” instead of “treatment-naive” risk. This might lead to incorrect risk predictions and even suboptimal treatment decisions, as demonstrated in Xu et al. and in simulations by others (3, 14). As such, it is helpful to first clarify the estimand that is being targeted, even in prediction (15). Doing so provides clarity on the assumptions that are required for a proposed method to provide accurate predictions of the required estimand, and indeed, clarity on exactly what is meant by “baseline risk,” which is more nuanced than it first might seem.

On top of providing the machinery to address issues such as treatment drop-in, the strengths of causal inference, if combined with established practices of CPM development, open a wide range of opportunities.

First, causal reasoning allows us to clarify some of the so-called paradoxical findings that are sometimes observed in CPMs. An oft-quoted example was discussed by Caruana et al. (16), in which patients with pneumonia and asthma in a hospital setting had better outcomes than those with pneumonia only, because of a policy that saw patients with asthma in addition to pneumonia directly admitted to the intensive care unit; this was originally ignored when building the CPM. Similarly, use of causal inference will help to overcome the more subtle, yet pernicious, challenge of risk-factor associations being attenuated because treatment is received differentially according to the value of the risk factor (17, 18). Although this is not necessarily an issue for the accuracy of a prediction model, it can greatly reduce the face validity and acceptability (17). Causal reasoning allows us to explain such paradoxical associations and lower barriers for the implementation of a prediction model in clinical practice, and make models more explainable. Causal inference methods can also help to examine the “counterfactual fairness” of a CPM and identify unwanted discriminatory behavior (19).

Second, it can allow better generalizability of a CPM. Particular interventions or policies might be present in the setting in which a CPM is developed but might not exist in a setting where the model is to be used—data-set shift (20). Dickerman and Hernán (21) give an example where individuals with severe heart failure are likely to receive a heart transplant, thus reducing their risk of death, in the population in which a CPM is developed. This CPM will perform poorly in a setting where the availability of heart transplants is low. Causal prediction can be used to issue predictions depending on the availability of heart transplants; thus, a causal CPM could be generalized to a setting regardless of this. Moreover, it can make explicit the complex feedback loop that arises when the use of a CPM itself changes outcome risks (which, indeed, is likely to be a sign of success of the CPM!) and thus allow the CPM to generalize over time. Once a CPM is deployed, it should be regularly updated (22), yet causal reasoning is needed to explicitly model the relationship between the baseline risk and the actions taken in response to that risk as estimated by the CPM (23).

Third, it introduces the possibility of calculating hypothetical risk under a range of possible interventions and therefore directly informing a decision about which intervention(s) to choose (24). It is too tempting at present for end-users of CPMs to do this, incorrectly, by modifying inputs to the CPM. For example, one might use QRISK (25) to estimate the impact of a weight loss intervention on a patient’s cardiovascular risk by entering a lower body mass index into the calculator. This is clearly wrong (26), but we have anecdotal evidence that this occurs and hypothesize that the practice is widespread. Enriching CPMs with the causal machinery needed to do this correctly could therefore have substantial benefits in terms of optimizing decisions supported by CPMs. This might be considered “pure” causal inference (12); however, we believe the additional considerations when developing and validating CPMs are also useful, such as optimizing for estimating absolute risk, ensuring that models are pragmatic to implement, and supporting clinical decisions on an individual level.

Finally, it clarifies the assumptions upon which these CPMs are relying, in terms of comparability of the development and deployment populations.

Indeed, rather than asking when causal reasoning can help with prediction, one might instead ask when it is not useful. Whenever a decision to intervene is made that can potentially affect future outcomes, and therefore predicted risk, causal approaches will be beneficial. Not all medical decisions fall into this category; some decisions can be made because of a particular risk, and not to affect it, and in these cases causal inference would not be required. For example, in a palliative care setting it might be useful for patients and their families to know the predicted outcomes.

Despite the clear advantages, the use of causal prediction is not widespread. This is because there are substantial challenges to be overcome before it can be implemented effectively.

First, validation is a major challenge. CPMs are usually validated in a test data set by considering the accuracy, calibration, and discrimination of the predictions issued. This relies on factual data: the availability of predictors and the corresponding observed outcomes. Validation of potential outcomes requires a different solution, because the outcomes are, by definition, not observed. Where the hypothetical scenario is a population in which no one receives treatment, one solution might be to validate the model using historical or geographically different data, where the treatment is not prevalent. However, such data might not be available and might differ in other ways from the current target population for the CPM. Xu et al. (6) attempt to overcome this by generating counterfactual treatment-naive survival times by adjusting the factual survival times according to the assumed risk reduction conferred by statins. However, the validation is then, partly at least, a self-fulfilling prophecy, because both the fitted model and the validation data use the same adjustment for the assumed causal effect of statins. Therefore, only the “prediction” part of the model is validated, under the assumption that the causal adjustment for the effect of statins is correct. Approaches where adjusted or synthetic outcomes are generated for validation data therefore require further scrutiny. Validation therefore remains perhaps the most pressing challenge to overcome before use of causal CPMs can become more widespread (24).

Second, Xu et al. (6), and most other literature on this topic, have considered only single interventions in isolation. Of course, the reality is far more complex than this. Even to define a treatment-naive prediction requires the consideration of all relevant interventions that are operating in a particular setting. For example, alongside prescribing a statin, a physician might recommend a range of lifestyle interventions, such as increased exercise, changes in diet, and quitting smoking, all of which could be considered as interventions. This is challenging both to elicit and to model effectively.

Third, because causal predictions involve potential outcomes, and are by definition out-of-sample predictions, we must be cautious about extrapolation. Causal approaches typically require more data: For example, using causal prediction to make a treatment decision using inverse probability weighting requires that we observe at least some patients with the characteristics of interest receiving both “actions” under consideration. In Xu et al. (6), the causal effect was instead estimated using external clinical trial data.

These challenges might lead one to conclude that causal prediction is simply too challenging and should not be considered. We disagree, primarily because there is a clinical need for such predictions. To fill the void, existing (factual) CPMs are already being used as if they provide causal predictions. This can lead to unsubstantiated conclusions and even incorrect clinical decisions being made. Therefore, there is an urgent need for causal prediction to provide clarity and correctness to the use of CPMs in this way.

In conclusion, CPMs are often interpreted and used as if their predictions refer to causal scenarios, and indeed used to compare risk under different hypothetical interventions. Discouraging such practice is likely to be unhelpful, and risks undermining the important progress made in improving the reporting and robust deployment of prediction models recently achieved, for example with the TRIPOD statement (27). A much more fruitful direction is likely to be enriching CPMs with the machinery needed to correctly (and with awareness of the assumptions required) provide the causal predictions that are really of interest to decision-makers. The approach of Xu et al. is an important step in upgrading the machinery of CPMs toward that goal, although extensions that account for both the uncertainty and (lack of) generalizability in the causal estimates are required. We would recommend the Xu et al. approach (6) be used alongside complementary approaches that estimate the intervention effects from the observational data (3, 9) to ensure maximum robustness.
